# Supplementary material for: HIVprotI: an integrated web based platform for prediction and design of HIV proteins inhibitors
Source: J Cheminform. 2018 Mar 9;10:12. doi: 10.1186/s13321-018-0266-y (PMC5845081; doi:10.1186/s13321-018-0266-y)
Supplement: Supplementary file 2 — Additional file 2. Source code of HIVProtI web server. [file 13321_2018_266_MOESM2_ESM.zip › HIVprotI_Source-code/hivprotI/weka-3-6-6/doc/weka/classifiers/UpdateableClassifier.html]

UpdateableClassifier


---


|  |  |  |
| --- | --- | --- |
| **Overview** \| **Package** \| **Class** \| **Tree** \| **Deprecated** \| **Index** \| **Help** \| **Weka's home** | |  |
| **PREV CLASS**   NEXT CLASS | **FRAMES**    **NO FRAMES**     **All Classes** |  |
| SUMMARY: NESTED \| FIELD \| CONSTR \| METHOD | DETAIL: FIELD \| CONSTR \| METHOD |  |


---


## weka.classifiers Interface UpdateableClassifier

**All Known Implementing Classes:**: AODE, AODEsr, DMNBtext, IB1, IBk, KStar, LWL, NaiveBayesMultinomialUpdateable, NaiveBayesUpdateable, NNge, RacedIncrementalLogitBoost, SPegasos, Winnow

---

``` public interface UpdateableClassifier ```

Interface to incremental classification models that can learn using
one instance at a time.

**Version:**
:   $Revision: 1.5 $

**Author:**
:   Eibe Frank (eibe@cs.waikato.ac.nz)

---

| **Method Summary** | |
| --- | --- |
| `void` | `updateClassifier(Instance instance)`             Updates a classifier using the given instance. |

**Method Detail**

### updateClassifier

```
void updateClassifier(Instance instance)
                      throws java.lang.Exception
```

:   Updates a classifier using the given instance.

    :   **Parameters:**: `instance` - the instance to included **Throws:**: `java.lang.Exception` - if instance could not be incorporated successfully


---


|  |  |  |
| --- | --- | --- |
| **Overview** \| **Package** \| **Class** \| **Tree** \| **Deprecated** \| **Index** \| **Help** \| **Weka's home** | |  |
| **PREV CLASS**   NEXT CLASS | **FRAMES**    **NO FRAMES**     **All Classes** |  |
| SUMMARY: NESTED \| FIELD \| CONSTR \| METHOD | DETAIL: FIELD \| CONSTR \| METHOD |  |


---
